# The Effect of Incorporating an Exergame Application in a Multidisciplinary Weight Management Program on Physical Activity and Fitness Indices in Children with Overweight and Obesity

**DOI:** 10.3390/children9010018

**Published:** 2021-12-29

**Authors:** Rotem Kahana, Shai Kremer, Merav Dekel Dahari, Einat Kodesh

**Affiliations:** 1 Maccabi Healthcare Services, Southern Region, Beer-Sheva 8489312, Israel; kahana_r@mac.org.il (R.K.); karm_sh@mac.org.il (S.K.); dekel_me@mac.org.il (M.D.D.); 2 Physical Therapy Department, University of Haifa, Haifa 3498838, Israel

**Keywords:** exergame, smartphone, obesity, fitness, motivation, weight, health behavior

## Abstract

Children with overweight/obese (OW/OB) have low physical activity (PA) levels and excessive daily screen times. Although access to personal smartphones may complicate restricting sedentary screen time, these devices may be used to promote PA and improve fitness. Therefore, we aim to examine the impact of incorporating an exergame application (APP) into an existing weight management program on BMI, physical activity, fitness levels, and attitude toward PA among OW/OB children. Seventy-nine children (51% girls), median age 10 years, completed an established 5-month weight management program. The intervention included structured PA sessions (2/week), nutritional, and behavioral counseling. An exergame app was installed on the smartphones of the intervention group (APP, *n* = 32). BMI, physical fitness, PA level, and attitudes toward PA were assessed before and after the intervention. BMI decreased (*p* < 0.0001) in both groups by 0.67 kg/m^2^ (Q1, Q3: −1.36–0.12). There were improvements in more fitness components in the APP group than controls, with significantly greater improvements in aerobic fitness (*p* = 0.038), speed and agility (*p* = 0.01), and leg strength endurance (*p* = 0.05) compared to controls. PA levels increased similarly in both groups during the intervention period. The incorporation of an exergame application leads to more significant improvements in fitness components. These findings support the use of exergame apps to improve fitness in OW/OB children.

## 1. Introduction

Over the last 30 years, childhood obesity has become a global epidemic [1,2] despite the well-known links between obesity, chronic diseases, and health problems [3]. Its prevalence in children and adolescents is rising [4]. According to the Organization for Economic Cooperation and Development (OECD), 26% of boys and 24% of girls under the age of 20 are overweight or obese (BMI ≥ 85th percentile) [5]. The rate of overweight children increased from 20.5% to 31.4% across 35 OECD countries between 1990 and 2016. From the 2011HBSC survey [6,7] of Israeli children and adolescents in 6–10th grade: the prevalence range for overweight (i.e., 85% ≤ BMI < 95%) boys was 13.7% to 16% and for obese (i.e., BMI ≥ 95%) boys was 13.7% to 17.6%. Thus, approximately 30% of the boys were above the recommended range. Among 6–10th-grade girls, 8.9–12.8% were overweight and 4.8–8% were obese, such that approximately 15% are above recommended range. About one in four children in grades 7–12 at school are overweight or obese [8].

While numerous factors have been shown to affect children’s physical activity (PA), such as parental and peer PA levels, ecological models show that built-up environments, the lack of parks, recreational facilities, access to sidewalks and paths for walking and cycling reduce opportunities for (PA), thereby promoting a sedentary lifestyle [9,10]. In addition, the parental and child perceptions of accessibility and safety of exercise facilities for children were found to predict PA and body fat in children [11].

The World Health Organization recommends at least 60 min of moderate to high-intensity aerobic exercise daily, 2–3 resistance workouts per week, and encouragement of children to be as active as possible during the day [12].

The Cochrane review by Waters et al. [13] analyzed studies on a total of 27,946 children (aged 6–12 years) participating in overweight intervention programs. They noted that few programs caused a decrease in BMI. Considering the different durations, types of intervention, and degree of parental involvement in the reviewed programs, it is difficult to isolate the factors essential for success [13]. However, multidisciplinary approaches combining dietary interventions increased PA, and behavioral strategies such as reducing screen time have significantly lowered BMI [14,15]. Overweight and obese adolescents have lower physical fitness levels and capabilities than their healthy peers. Physical fitness, determined by strength and cardiovascular abilities, are important health markers, which may predict risk for cardiovascular diseases [16,17,18]. Hence, fitness indicators such as aerobic capability and muscle strength are an important part of program evaluation and can be used to help establish goals for interventions in children.

Technological developments influence how children spend their leisure time [19]. According to the American Academy of Pediatrics (AAP), children aged 8–18 years spend about seven hours a day watching TV or using computers and mobile phones [20]. Restricting technology use has been recommended to create a more active lifestyle [21]. However, smartphones have become very popular among youth. For example, about 31% of American children aged 8–10 years, 69% aged 11–14 years, and 85% aged 15–18 years have their own smartphone [20]. In 2017, 89.5% of people over age three had smartphones in South Korea [22,23]. Thus, reducing screen time becomes challenging.

A strategy that employs and takes advantage of the available technologies to promote health [24,25,26,27] may be useful in promoting PA [26], and various programs worldwide use available technology to create active and physically challenging games (exergames) that encourage PA [28] Exergames have been found to promote physical activity, specifically among children with chronic conditions [29,30]. Incorporating such games in programs for children with overweight or obesity (OW/OB) may result in a greater reduction in BMI, higher adherence to PA [31], improved self-esteem [32], and a reduction in sedentary screen time [33]. However, as concluded by a recent meta-analysis aimed to assess the possible role of exergames in reducing weight-related outcomes among OW/OB children, clear evidence about the effectiveness of exergames in determining weight loss in pediatric obesity is still lacking [26]. In addition to the established benefits of exergames, motivation to participate in PA is necessary to create lasting behavioral changes. Enjoyment, interest, self-efficacy, and gratification contribute to internal motivation [34,35]. Exergames are considered by children as fun, interesting, visually attractive, interactive, challenging, and satisfying [36], which may increase activity. Moreover, exergames divert the child’s attention from how their bodies are perceived by others during PA to the motor activity of the game itself [37]. Sensors can assess exercise intensity by incorporating heart rate monitors [38], accelerometers [39], GPS technologies [40], and systems that enable social interaction and may also increase enjoyment.

The advantages of an exergame smartphone app include its relatively low cost, accessibility, the ability to regularly update online, and its perception as attractive by children. However, the benefits of using exergaming for weight management in children and adolescents remain unknown. In a previous meta-analysis aimed to determine the impact of exergaming on weight loss in this population, no differences were found compared to controls [41].

In this study, we aimed to examine the impact of integrating a smartphone PA exergame application into an established weight management program for children with OW/OB on various outcomes including, BMI, physical fitness, PA participation, and attitudes toward PA, in order to determine whether exergame apps can be used as a tool to promote additive changes in behavior and increase physical activity. We hypothesized that integrating an exergame app into the program would promote changes in behavior and increase physical fitness.

## 2. Materials and Methods

### 2.1. Study Design and Procedure

**Figure 1:**
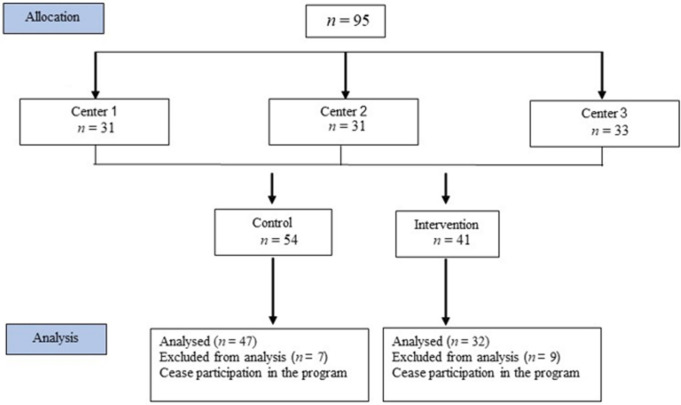
Study flow diagram.

The study was approved by the Ethics Committee of the Health Organization number 0067-16-BBL. Prior to the intervention and after a detailed explanation, ninety-five participants and their parents signed an informed consent form. Participant BMI was ≥85th percentile for age and sex, with no medical conditions restricting PA, and all participants had access to smartphone. Maccabi Health Organization provided a 5-month weight management program for children and adolescents, and 3 centers were involved in the current study. In two centers, the exergame APP was incorporated in rotating consecutive cycles of the program. That is, cohorts were assigned consecutively to the control group, then to the app (intervention) group. In the third center, the exergame was not incorporated, and those treated at this center were included in the control group (see Figure 1).

### 2.2. Participants

All participants were enrolled in the five-month weight management program in one of the 3 Maccabi centers after referral from their family physician or dietitian. Out of the 95 children registered in the program and agreeing to participate, data from 16 individuals (7 controls and 9 app group) were excluded from analysis due to failure to complete the program. Data from 79 children were included in the final analysis.

### 2.3. Interventions Protocol

The program included twice-weekly, hour-long group PA sessions led by a fitness trainer, 3–5 group nutritional consultation sessions with a dietitian, 12–13 parent group sessions with a dietitian and a social worker, a healthy cooking workshop, and a combined children-parents PA session. In addition, each child had 2–3 one-on-one sessions with a dietitian and two consultations with a physiotherapist to set individual PA goals.

The PA sessions typically included cardiovascular endurance, muscle strength, speed and agility, coordination, balance, and flexibility exercises. Participants had approximately 50 contact hours during the program.

The intervention group (APP) took part in the program described above and had unrestricted access to PA apps “Just Dance Now” and “Motion Sports”, which were installed on their smartphones. One of the weekly PA sessions included a group activity using an app. The “Just Dance Now” app consists of various dance moves accompanied by music, and points are awarded for following the movements of a virtual figure. In the “Motion Sports” app, four sports activities (swimming, running, skiing, and soccer) are available. Motion technology detects the players’ movements and their moves compared with those of the on-screen characters. In addition, there are items to collect during the game to boost scores.

All participants received a weekly text message encouraging them to be active for at least an hour a day. The APP group was also encouraged to use the apps on their smartphone. Participants were asked to record the number of minutes they were active after each activity and send a message every Sunday morning using the application “WhatsApp” with total PA time and intensity (1—easy, 2—medium, 3—hard).

At the beginning of the program, anthropometric measurements were taken, and self-report questionnaires to evaluate weekly PA levels and attitudes toward physical activity were completed by the children, with help provided by the physical therapist or parent, if needed. The participants were familiarized with the fitness test procedures one week prior to assessment. Assessment included tests of speed and agility, strength and coordination, and aerobic components, which were performed at the beginning and end of the intervention program. Attendance at group sessions was recorded as an indicator of compliance.

### 2.4. Measurements

All measurements were taken at the beginning of the program and repeated at the end of the five-month intervention, in the same order. No adverse events related to the intervention were reported.

#### 2.4.1. Anthropometric Measurements

Anthropometric measurements were taken by the same clinical staff member at each participating center. Participants were barefoot, weight (kg) and height (cm) were measured using a standard scale and a stadiometer (Health O Meter), and BMI was calculated. BMI percentage was defined by the Cole criteria [42].

#### 2.4.2. Fitness Tests

All the fitness tests included in the study were field-based and easy to conduct, allowing the assessment of a variety of fitness components. Fitness tests were performed by the same physical therapist (R.K.) and fitness instructor for all groups.

Agility was evaluated using the Shuttle Run 4 × 10 m, which has been found to be a valid and reliable test for children and adolescents [43]. Participants were asked to run 10 m in one direction and then return as quickly as they could four times; the time for completing the entire run was recorded.

Leg power performance was evaluated using the two-legged Standing Long Jump Test [44]. The participants were instructed to jump as far as possible twice. The longest jump distance was recorded and used for analysis. This test is considered a valid and reliable fitness test in children and adolescents [44,45].

Muscle endurance for lower extremities was tested using the Wall Sit Test [46]; during the test, the participants squatted with their backs against the wall (hips and knees at 90°), and hands crossed over their chest, then lifted and held one foot 5 cm off the ground for as long as possible. The sum of 2 times per leg was recorded and used for analysis.

Hand-eye coordination was assessed using the Hand Wall Toss Test [47]. The children were asked to stand 2 m from a wall and throw and catch a tennis ball as many times as possible in 30 s. This test is valid and reliable as part of the battery of tests for child movement assessment [47].

Maximal grip force was measured using a hand dynamometer (Jamar) [43] with the elbow flexed at 90 degrees. The test was repeated twice in each hand; the average score was calculated and used for analysis. High-reliability coefficients have been reported for this test in children 6–12 years old [48].

Aerobic fitness: was assessed using the 20 m Yo-Yo Test, as described previously by Léger [49]. Total distance run was calculated. The Yo-Yo Test is considered to be reproducible and may be used as an indicator of aerobic fitness for children under 10 years of age [50].

#### 2.4.3. Questionnaires

All questionnaires were completed by the children, with assistance provided by the physical therapist or parent, as needed.

Physical Activity Questionnaire for Children (PAQ-C) was used to evaluate weekly PA levels. The PAQ-C includes 9 items. Children were asked to recall their participation in activities over the last 7 days. Value from 1 to 5 was given for each of the 9 items. The mean value of these 9 items is the final PAQ-C activity summary score [51]. PAQ-C has been reported to have excellent content validity, acceptable inter-item reliability, and a moderate to suitable strength of inter-rater agreement [52].

Attitude toward physical activity was evaluated using the “Behavioral Regulation in Exercise Questionnaire” (BREQ-2), previously used with adolescents [53]. The questionnaire comprises 19 items relating to five motivation types: amotivation (e.g., “I don’t see the point in being physically active”), external regulation (e.g., “I am physically active because other people say I should”), introjected regulation (e.g., “I feel guilty when I’m not physically active”), identified regulation (e.g., “I’m physically active because I value the benefits of physical activity”) and intrinsic motivation (e.g., “I’m physically active because it’s fun). Each item is measured on a five-point Likert scale, from 0 (‘Not true for me’) to 4 (‘Very true to me’). The mean of the 5 subscales is calculated for each motivation type separately. The Relative Autonomy Index (RAI) is calculated by weighting each subscale and summing the weighted scores: (amotivation multiplied by −3) + (external regulation multiplied by −2) + (introjected regulation multiplied by −1) + (identified regulation multiplied by 2) + (intrinsic regulation multiplied by 3). The minimum score for the RAI is −24, and the maximum score is +20. Higher scores for the RAI indicate more autonomous motivation, whereas lower scores indicate less autonomous motivation. The questionnaire has been validated for use in obese pediatric populations [54].

### 2.5. Statistical Analyses

Statistical analyses were performed using SAS (version 9.4). Normality was tested using the Shapiro–Wilk test, which indicated that our outcomes were not normally distributed. Therefore, data are expressed as median (Q1, Q3). To test the differences within the group (pre, post) signed rank test was used. In order to evaluate the effects of implementing the exergame app, delta (post-pre) was calculated for each variable, and the Wilcoxon test was used to examine differences between groups. Statistical significance was set at *p* < 0.05.

## 3. Results

A total of 79 children (40 girls (51%); 39 boys (49%)) completed the study. There were 32 (16 girls (50%); 16 boys (50%) children in the APP group and 47 (24 girls (51%); 23 boys (49%)) children in the control group. Age was significantly different between the groups; the APP group participants were younger (*p* = 0.05) with a median age of 9 years (Q1, Q3: 8.0, 10.0) compared to the control group with a median age of 10 years (Q1, Q3: 8.0, 11.0).

### 3.1. Anthropometric Measures

Anthropometric measures are presented in Table 1. No differences between the groups were found in the anthropometric measures pre and post intervention (see Table 1). After the intervention, body mass was maintained in both groups, while height increased by approximately 2 cm and BMI decreased by 0.67 kg/m^2^ (Q1, Q3: −1.36–0.12) kg/m²) for the whole cohort (S = 934.5, *p* < 0.0001) (APP S = 129, *p* < 0.0090, Con S = −377.5 *p* < 0.0001). No difference was found in BMI% change between the groups, while median BMI% remained high (99%), and only 15 participants (6 from the APP group) demonstrated a decrease in BMI percentile.

### 3.2. Fitness Components

Table 2 presents fitness results pre and post intervention and the delta between these time points (post-pre). At pre intervention, measurements the control group was achieved higher aerobic (distance at the Yo-Yo Test) and higher grip force compared to the APP group (Table 2).

APP group improved in most fitness tests: Aerobic component tested by Yo-Yo running distance (*p* < 0.0001), speed and agility tested by 4 × 10 m run (*p* < 0.0001), leg strength endurance measured by wall sit-up test (*p* = 0.0002), handgrip using a dynamometer (*p* = 0.0029), hand-eye coordination measured by Hand Wall Toss Test (*p* < 0.0001).

The control group only showed improvement in the 4 × 10 m run (*p* = 0.0016) and Hand Wall Toss Test (*p* = 0.03). Comparison of the changes (deltas) following the 5-month intervention between the groups revealed that: Yo-Yo running distance, 4 × 10 m, and wall sit-up test improved to a greater extent in the APP group.

### 3.3. Attitude toward Physical Activity

Attitude toward PA was measured by BREQ-2. The pre-intervention score of the intrinsic regulation component was lower in the control group compared to the APP group (APP median 3.8 (Q1, Q3; 3.0, 4.0), control, median 3.0 (Q1, Q3; 2.3, 3.7), *p* = 0.013). The control group improved significantly in their RAI BREQ score (pre 9.8 (Q1, Q3; 4.5, 12.5) post 11.4 (Q1, Q3; 4.5, 12.5) S = 210 *p* = 0.01). The differences in the APP group (pre 10.3 (Q1, Q3; 5.3, 13.6) Post 11.3 (Q1, Q3; 7.3, 13.8) S = 37 *p* = 0.384) were not statistically significant. Comparison of the delta changes flowing the intervention revealed non-significant differences between the groups

### 3.4. Program Compliance and Self-Reported Physical Activity

Compliance: An attendance of 74.4% was observed in PA sessions, with no differences between the groups. During the program, APP reported higher training intensity (mean score of 2.2 points out of 3) than controls (1.88 points) (*p* = 0.046). The average weekly physical activity duration was 349 ± 168 min, with no difference between the groups. During the intervention period PAQ-C questionnaire score improved by 1.2 points (maximum score is 5 points) in both groups (APP, pre median score 2 (Q1, Q3; 1.7, 2.7) Post median score 3.3 (Q1, Q3; 3.0, 3.6) S = 205.5 *p* < 0.0001; control pre median 1.6 scores (Q1, Q3; 1.3,2.1) post median score 3.2 (Q1, Q3; 2.7, 3.9) S = 509.5 *p* < 0.0001).

## 4. Discussion

The multidisciplinary weight management program resulted in weight maintenance in both groups, with a decrease in BMI. However, the overall BMI percentile remained high. Incorporating the exergame app led to improvements in most of the tested physical fitness components, specifically aerobic, speed and agility, and leg muscle endurance. The integration of the app did not lead to a more significant decrease in BMI. Since body mass is expected to increase due to growth and development, the fact that BMI remained unchanged, or even slightly decreased, can be considered a success. The effectiveness of multidisciplinary programs in children with OW/OB was addressed in a meta-analysis, which concluded that there was often no decrease in BMI [55]. For example, Hoffman [56] compared standard treatment by a pediatrician with a community-based PA intervention that included additional weekly access to PA classes for six months in children aged 5–11 years, with BMI ≥ 95%, and reported that BMI did not decrease in either group [56]. On the other hand, Nemet et al. (2014) reported decreases of 1.2 kg and 1.3 kg/m^2^ in BMI after three months of a multidisciplinary program in 10-year-old children [57]. The latter program included three weekly PA sessions and a customized menu consisting of 1200–2000 kcal daily or a 15–30% reduction in daily reported consumption. In the current study, the children participated in two weekly exercise sessions and had a general menu with no carbohydrate restrictions. It is important to mention that a significant decrease in weight is needed in order to demonstrate a reduction in BMI when the BMI percentile is >95. Such a significant weight decrease was not the current study’s purpose and may not even be recommended in children [58].

Staiano et al. (2017) examined the influence of exergaming on body composition in adolescent girls with OW/OB. In agreement with our findings, they showed that 12 weeks of self-selected dance-based exergames reduced subcutaneous abdominal adipose tissue (measured by MRI), attenuated fat gain but was not sufficient to decrease BMI percentile [59]. However, 24 weeks of intervention with a home-based exergame coupled with telehealth fitness consulting [60] improved children’s (10–12 years of age) BMI z-score and physical activity level compared to the control group who were asked to maintain their normal level of physical activity. One potential explanation for the relative success of this intervention is the telehealth fitness coach that increased motivation to exercise and promoted self-efficacy.

The association between poor physical fitness and obesity in children has been reported extensively [61]. We found that integration of the exergame app into the program improved some fitness components: aerobic endurance, leg muscle endurance, and 4 × 10 m running speed compared to controls. This improvement may be related to the higher training intensity reported by the children in this group. Physical activity improves lifestyle and health, maintains weight loss, and contributes to self-confidence and a sense of competence [62]. Furthermore, PA during childhood helps to establish a pattern of participation in PA later in life [63].

The PAQ-C questionnaire was designed to assess physical activity levels [64], and despite extensive use, no norms have been determined to quantify the degree of change in reported activity levels. For example, Chen et al. (2008) defined PAQ ≤ 2 as low activity level, 3 ≥ PAQ > 2 as medium activity level, and PAQ > 3 as highly active [65], based on this, the children in our study moved from low activity levels to high activity levels at the end of the intervention, regardless of the study group.

Motivation to participate in PA is one of the most important factors for predicting current and future PA levels [66]. According to self-determination theory, three factors influence the decision to participate in PA: lack of motivation, external motivation, and internal motivation, and there are three psychological needs that an intervention must respond to in order to enable behavioral change: autonomy and choice, sense of success and a sense of belonging and acceptance [67]. Children with overweight and obesity commonly demonstrate higher levels of external motivation and low levels of internal motivation to participate in PA [68]. Since exergames reportedly increase the feeling of autonomy and internal motivation [69].

We assumed that the incorporation of the App would enhance internal motivation to exercise, but we found no improvements. Total BREQ score increased following the intervention and only reached statistical significance in the control group. Compared to the study by Verloigne [54] that used the same questionnaire in children with obesity that participated in an obesity treatment program, our cohort was more motivated at the beginning of the study (9 vs. 5); however, both studies demonstrated an improvement of approximately 2 points.

Our research is not free of limitations. The APP group had greater improvements in certain components of physical fitness. However, it is possible that a more sophisticated app that quantifies user effort, provides feedback, and encourages communication between users may yield even better results. There were no changes in BMI percentile in our participants. It is possible that if more sensitive measurements, such as waist-to-height ratio or waist circumference, were used, we would have been able to detect the effects of the intervention. In addition, PA behavior and intensity were not measured objectively and may be over-estimated by self-report; nevertheless, this potential bias was similar in both groups. Finally, the degree of app use and user satisfaction were not assessed. Notably, the study was conducted within a real-life clinical setting rather than a lab-based design. Thus, it can be argued to have ecological validity.

## 5. Conclusions

The multidisciplinary weight management program resulted in weight maintenance and improved physical activity behavior and fitness. The integration of an exergame app into an established multidisciplinary program was advantageous, as noted by improved results in physical fitness.

Since the use of smartphones has increased, and in view of the fact that appropriate strategies for increasing physical activity among children with OW/OB are needed, the use of more sophisticated applications that combine effort information, feedback, and interpersonal communication should be examined.

## Figures and Tables

**Table 1:** Participant’s physical characteristics before and after the weight management program.

Variable	APP (*n* = 32)			Control (*n* = 47)		
	Pre Median (IQR)	Post Median (IQR)	Within-group *p*-value	Pre Median (IQR)	Post Median (IQR)	Within-group *p*-value *
Height (cm)	141.5 (18.50)	142.0 (21.10)	<0.0001	147.0 (15.00)	148.3 (14.40)	<0.0001
Weight (kg)	46.8 (18.20)	47.5 (21.40)	NS	55.5 (23.30)	55.6 (23.50)	NS
BMI (kg/m²)	25.4 (4.19)	23.3 (5.24)	0.009	26.0 (4.20)	25.3 (4.44)	<0.0001
BMI percentile	99 (0.00)	99 (2.00)	NS	99 (0.00)	99 (2.00)	NS

IQR—interquartile range; NS—not significant; * signed rank within group.

**Table 2:** Fitness tests pre and post intervention and the change post intervention (∆ = post-pre) in both groups (APP; control).

	APP *n* = 32			Control *n* = 44			Between Group #
Variable	Pre Median (IQR)	Post Median (IQR)	∆ Median (IQR)	Pre Median (IQR)	Post Median (IQR)	∆ Median (IQR)	∆ *p*-value
Distance Yo-Yo Test (m)	200.0 # (100.00)	240 ** (110.0)	60.0 (100.00)	240.0 (160.00)	240.0 (170.00)	20.0 (160.00)	0.038
4 × 10 m (sec)	13.5 (3.60)	12.6 ** (2.62)	−0.9 (1.13)	14.1 (2.76)	13.5 * (1.87)	0.3 (1.44)	0.010
Wall Sit-Up (sec)	24.2 (26.68)	41.4 ** (26.61)	14.9 (22.26)	25.0 (19.35)	29.3 (26.10)	3.8 (17.04)	0.050
Standing Long Jump (cm)	101.5 (21.00)	100 (21.50)	−5.0 (21.50)	111.0 (26.00)	105.0 (21.00)	4 (24.00)	0.37
Hand Grip (kg)	12.5 # (11.75)	13.8 * (12.75)	1.0 (2.25)	16.50 (7.00)	17.0 (6.75)	0.5 (3.00)	0.40
Hand Wall Toss Test (# catches)	8 (8.00)	12.5 ** (11.50)	3.0 (7.50)	7.0 (16.00)	11.0 * (10.00)	2.0 (5.00)	0.18

IQR—interquartile range; ∆ = post-pre change intervention; * signed rank within group *p* ≤ 0.005, ** <0.001; # Wilcoxon two-sample test between group *p* ≤ 0.05.

## Data Availability

The data presented in this study are available on request from the corresponding author.

## References

[1] Lobstein T., Jackson-Leach R., Moodie M.L., Hall K.D., Gortmaker S.L., Swinburn B.A., James W.P.T., Wang Y., McPherson K. (2015). Child and adolescent obesity: Part of a bigger picture. Lancet.

[2] Sahoo K., Sahoo B., Choudhury A.K., Sofi N.Y., Kumar R., Bhadoria A.S. (2015). Child-hood obesity: Causes and consequences. J. Fam. Med. Prim. Care.

[3] Barlow S.E. (2007). the Expert Committee: Expert committee recommendations on the as-sessment, prevention, and treatment of child and adolescent overweight and obesity: Summary report. Pediatrics.

[4] Dietz W.H. (2011). Reversing the tide of obesity. Lancet.

[5] Tremblay M.S., Barnes J.D., González S.A., Katzmarzyk P.T., Onywera V.O., Reilly J.J., Tomkinson G.R. (2016). Global Matrix 2.0: Report Card Grades on the Physical Activity of Children and Youth Comparing 38 Countries. J. Phys. Act. Health.

[6] (2011). Health Behavior in School-Aged Children: WHO Collaborative Cross-National Survey/Study (HBSC) 2010–2011.

[7] Ministry of Health (2020). Prevention and Treatment of Obesity, A Subcommittee of the Health Behaviors Committee.

[8] Content Source: Division of Nutrition, Physical Activity, and Obesity, National Center for Chronic Disease Prevention and Health Promotion Control. https://www.cdc.gov/obesity/data/childhood.html.

[9] Ogden C., Carroll M.D., Curtin L.R., Lamb M.M., Flegal K.M. (2010). About childhood obesity. JAMA.

[10] Smith M., Hosking J., Woodward A., Witten K., MacMillan A., Field A., Baas P., Mackie H. (2017). Systematic literature review of built environment effects on physical activity and active transport—An update and new findings on health equity. Int. J. Behav. Nutr. Phys. Act..

[11] Horodyska K., Boberska M., Knoll N., Scholz U., Radtke T., Liszewska N., Luszczynska A. (2018). What matters, parental or child perceptions of physical activity facilities? A prospective parent-child study explaining physical activity and body fat among children. Psychol. Sport Exerc..

[12] Bull F.C., Al-Ansari S.S., Biddle S., Borodulin K., Buman M.P., Cardon G., Carty C., Chaput J.-P., Chastin S., Chou R. (2020). World Health Organization 2020 guidelines on physical activity and sedentary behaviour. Br. J. Sports Med..

[13] Waters E., de Silva-Sanigorski A., Hall B.J., Brown T., Campbell K.J., Gao Y., Armstrong R., Prosser L., Summerbell C.D. (2011). Interventions for preventing obesity in children. Cochrane Database Syst. Rev..

[14] Peirson L., Fitzpatrick-Lewis D., Morrison K., Ciliska D., Kenny M., Ali M.U., Raina P. (2015). Prevention of overweight and obesity in children and youth: A systematic review and meta-analysis. CMAJ Open.

[15] Visscher T.L.S., Kremers S.P.J. (2015). How can we better prevent obesity in children?. Curr. Obes. Rep..

[16] Garcia-Hermoso A., Cavero-Redondo I., Ramirez-Velez R., Ruiz J.R., Ortega F.B., Lee D.C., Martinez-Vizcaino V. (2018). Muscular Strength as a Predictor of All-Cause Mortality in an Apparently Healthy Population: A Systematic Review and Meta-Analysis of Data From Approximately 2 Million Men and Women. Arch. Phys. Med. Rehabil..

[17] Garcia-Hermoso A., Esteban-Cornejo I., Olloquequi J., Ramirez-Velez R. (2017). Cardi-orespiratory Fitness and Muscular Strength as Mediators of the Influence of Fatness on Academic Achievement. J. Pediatr..

[18] Ortega F.B., Ruiz J.R., Castillo M.J., Sjostrom M. (2008). Physical fitness in childhood and adolescence: A powerful marker of health. Int. J. Obes..

[19] Karsten L. (2005). It all used to be better? Different generations on continuity and change in urban children’s daily use of space. Child. Geogr..

[20] Rideout V.J., Foehr U.G., Roberts D.F. (2010). Generation M^2^: Media in the Lives of 8-to 18-Year-Olds.

[21] Davis M.M., Gance-Cleveland B., Hassink S., Johnson R., Paradis G., Resnicow K. (2007). Recommendations for prevention of childhood obesity. Pediatrics.

[22] Choi D.J., Kim Y.S., Um N.R., Kim H.S. (2018). The Survey on Smartphone Overdependence [Internet]. Annual Report.

[23] Park J.H., Park M. (2021). Smartphone use patterns and problematic smartphone use among preschool children. PLoS ONE.

[24] Talbot T.B. (2011). Virtual reality and interactive gaming technology for obese and diabetic children: Is military medical technology applicable?. J. Diabetes Sci. Technol..

[25] Chen H., Sun H. (2017). Effects of Active Videogame and Sports, Play, and Active Recrea-tion for Kids Physical Education on Children’s Health-Related Fitness and Enjoyment. Games Health J..

[26] Valeriani F., Protano C., Marotta D., Liguori G., Romano Spica V., Valerio G., Vi-tali M., Galle F. (2021). Exergames in Childhood Obesity Treatment: A Systematic Review. Int. J. Environ. Res. Public Health.

[27] Zeng N., Gao Z. (2016). Exergaming and obesity in youth: Current perspectives. Int. J. Gen. Med..

[28] Song H., Peng W., Lee K.M. (2011). Promoting exercise self-efficacy with an exergame. J. Health Commun.

[29] Calvert S.L., Staiano A.E., Bond B.J. (2013). Electronic gaming and the obesity crisis. New Dir. Child Adolesc. Dev..

[30] Maddison R., Foley L., Ni Mhurchu C., Jiang Y., Jull A., Prapavessis H., Hohepa M., Rodgers A. (2011). Effects of active video games on body composition: A randomized con-trolled trial. Am. J. Clin. Nutr..

[31] Trost S.G., Sundal D., Foster G.D., Lent M.R., Vojta D. (2014). Effects of a pediatric weight management program with and without active video games: A randomized trial. JAMA Pediatrics.

[32] Gao Z., Chen S. (2014). Are field-based exergames useful in preventing childhood obesity? A systematic review. Obes. Rev..

[33] Christison A., Khan H.A. (2012). Exergaming for Health: A Community-Based Pediatric Weight Management Program Using Active Video Gaming. Clin. Pediatr..

[34] Guerra P.H., Nobre M.R., Silveira J.A., Taddei J.A. (2013). The effect of school-based physical activity interventions on body mass index: A meta-analysis of randomized trials. Clinics.

[35] Metcalf B., Henley W., Wilkin T. (2013). Republished research: Effectiveness of intervention on physical activity of children: Systematic review and meta-analysis of controlled trials with objectively measured outcomes (EarlyBird 54). Br. J. Sports Med..

[36] Gao Z., Zhang T. (2012). Children’s Physical Activity Levels and Their Psychological Cor-relates in Interactive Dance Versus Aerobic Dance. Med. Sci. Sports Exerc..

[37] Staiano A.E., Calvert S.L. (2011). Exergames for Physical Education Courses: Physical, Social, and Cognitive Benefits. Child Dev. Perspect..

[38] Wylie C.G., Coulton P. Mobile exergaming. Proceedings of the 2008 International Conference on Advances in Computer Entertainment Technology.

[39] Garde A., Umedaly A., Abulnaga S.M., Junker A., Chanoine J.P., Johnson M., An-sermino J.M., Dumont G.A. (2016). Evaluation of a Novel Mobile Exergame in a School-Based Environment. Cyberpsychol. Behav. Soc. Netw..

[40] Macvean A., Robertson J. iFitQuest: A school based study of a mobile location-aware exergame for adolescents. Proceedings of the 14th International Conference on Human-Computer Interaction with Mobile Devices and Services.

[41] Bochner R.E., Sorensen K.M., Belamarich P.F. (2015). The impact of active video gaming on weight in youth: A meta-analysis. Clin. Pediatr..

[42] Cole T.J., Bellizzi M.C., Flegal K.M., Dietz W.H. (2000). Establishing a standard definition for child overweight and obesity worldwide: International survey. BMJ.

[43] Ruiz J.R., Castro-Pinero J., Espana-Romero V., Artero E.G., Ortega F.B., Cuenca M.M., Jimenez-Pavon D., Chillon P., Girela-Rejon M.J., Mora J. (2011). Field-based fitness assessment in young people: The ALPHA health-related fitness test battery for children and adolescents. Br. J. Sports Med..

[44] Artero E.G., Espana-Romero V., Castro-Pinero J., Ruiz J., Jimenez-Pavon D., Apari-cio V., Gatto-Cardia M., Baena P., Vicente-Rodriguez G., Castillo M.J. (2012). Criterion-related validity of field-based muscular fitness tests in youth. J. Sports Med. Phys. Fitness.

[45] Thomas E., Petrigna L., Tabacchi G., Teixeira E., Pajaujiene S., Sturm D.J., Sahin F.N., Gomez-Lopez M., Pausic J., Paoli A. (2020). Percentile values of the standing broad jump in children and adolescents aged 6-18 years old. Eur. J. Transl. Myol..

[46] Eather N., Morgan P.J., Lubans D.R. (2013). Feasibility and preliminary efficacy of the Fit4Fun intervention for improving physical fitness in a sample of primary school chil-dren: A pilot study. Phys. Educ. Sport Pedagog..

[47] Dirksen T., De Lussanet M.H., Zentgraf K., Slupinski L., Wagner H. (2016). Increased Throwing Accuracy Improves Children’s Catching Performance in a Ball-Catching Task from the Movement Assessment Battery (MABC-2). Front. Psychol..

[48] Espana-Romero V., Artero E.G., Santaliestra-Pasias A.M., Gutierrez A., Castillo M.J., Ruiz J.R. (2008). Hand span influences optimal grip span in boys and girls aged 6 to 12 years. J. Hand Surg. Am..

[49] Leger L.A., Mercier D., Gadoury C., Lambert J. (1988). The multistage 20 metre shuttle run test for aerobic fitness. J. Sports Sci..

[50] Ahler T., Bendiksen M., Krustrup P., Wedderkopp N. (2012). Aerobic fitness testing in 6- to 9-year-old children: Reliability and validity of a modified Yo-Yo IR1 test and the Andersen test. Eur. J. Appl. Physiol..

[51] Benitez-Porres J., Lopez-Fernandez I., Raya J.F., Alvarez Carnero S., Alvero-Cruz J.R., Alvarez Carnero E. (2016). Reliability and Validity of the PAQ-C Questionnaire to Assess Physical Activity in Children. J. Sch. Health.

[52] Bervoets L., Van Noten C., Van Roosbroeck S., Hansen D., Van Hoorenbeeck K., Verheyen E., Van Hal G., Vankerckhoven V. (2014). Reliability and Validity of the Dutch Physical Activity Questionnaires for Children (PAQ-C) and Adolescents (PAQ-A). Arch. Public Health.

[53] Murcia J.A., Gimeno E.C., Camacho A.M. (2007). Measuring self-determination motivation in a physical fitness setting: Validation of the Behavioral Regulation in Exercise Question-naire-2 (BREQ-2) in a Spanish sample. J. Sports Med. Phys. Fit..

[54] Verloigne M., De Bourdeaudhuij I., Tanghe A., D’Hondt E., Theuwis L., Vansteen-kiste M., Deforche B. (2011). Self-determined motivation towards physical activity in adolescents treated for obesity: An observational study. Int. J. Behav. Nutr. Phys. Act..

[55] Wang Y., Cai L., Wu Y., Wilson R.F., Weston C., Fawole O., Bleich S.N., Cheskin L.J., Showell N.N., Lau B.D. (2015). What childhood obesity prevention programmes work? A systematic review and meta-analysis. Obes. Rev..

[56] Hoffman J., Frerichs L., Story M., Jones J., Gaskin K., Apple A., Skinner A., Arm-strong S. (2018). An Integrated Clinic-Community Partnership for Child Obesity Treatment: A Randomized Pilot Trial. Pediatrics.

[57] Nemet D., Levi L., Pantanowitz M., Eliakim A. (2014). A combined nutrition-al-behavioral-physical activity intervention for the treatment of childhood obesity–a 7-year summary. J. Pediatr. Endocrinol. Metab..

[58] Valerio G., Maffeis C., Saggese G., Ambruzzi M.A., Balsamo A., Bellone S., Ber-gamini M., Bernasconi S., Bona G., Calcaterra V. (2018). Diagnosis, treatment and preven-tion of pediatric obesity: Consensus position statement of the Italian Society for Pediatric Endocrinology and Diabetology and the Italian Society of Pediatrics. Ital. J. Pediatr..

[59] Staiano A.E., Marker A.M., Beyl R.A., Hsia D.S., Katzmarzyk P.T., Newton R.L. (2017). A randomized controlled trial of dance exergaming for exercise training in overweight and obese adolescent girls. Pediatr. Obes..

[60] Staiano A.E., Beyl R.A., Guan W., Hendrick C.A., Hsia D.S., Newton R.L. (2018). Home-based exergaming among children with overweight and obesity: A randomized clinical trial. Pediatr. Obes..

[61] Andersen J.R., Natvig G.K., Aadland E., Moe V.F., Kolotkin R.L., Anderssen S.A., Resaland G.K. (2017). Associations between health-related quality of life, cardiorespiratory fitness, muscle strength, physical activity and waist circumference in 10-year-old children: The ASK study. Qual. Life Res..

[62] Boreham C., Riddoch C. (2001). The physical activity, fitness and health of children. J. Sports Sci..

[63] Hills A.P., Andersen L.B., Byrne N.M. (2011). Physical activity and obesity in children. Br. J. Sports Med..

[64] Voss C., Ogunleye A.A., Sandercock G.R. (2013). Physical Activity Questionnaire for children and adolescents: English norms and cut-off points. Pediatr. Int..

[65] Chen S.R., Lee Y.J., Chiu H.W., Jeng C. (2008). Impact of physical activity on heart rate variability in children with type 1 diabetes. Childs Nerv. Syst..

[66] Lewis M., Sutton A. (2011). Understanding Exercise Behaviour: Examining the Interaction of Exercise Motivation and Personality in Predicting Exercise Frequency. J. Sport Behav..

[67] Ryan R.M., Deci E.L. (2000). Self-determination theory and the facilitation of intrinsic motivation, social development, and well-being. Am. Psychol..

[68] Hwang J., Kim Y.H. (2013). Physical activity and its related motivational attributes in adolescents with different BMI. Int. J. Behav. Med..

[69] Staiano A.E., Abraham A.A., Calvert S.L. (2013). Adolescent exergame play for weight loss and psychosocial improvement: A controlled physical activity intervention. Obesity.

